# App-Delivered Mindfulness Training to Reduce Anxiety in People with HIV Who Smoke: A One-Armed Feasibility Trial

**DOI:** 10.3390/ijerph20064826

**Published:** 2023-03-09

**Authors:** Patricia A. Cioe, Alexander W. Sokolovsky, Judson A. Brewer, Christopher W. Kahler

**Affiliations:** Center for Alcohol and Addiction Studies, Department of Behavioral and Social Sciences, Brown University School of Public Health, Providence, RI 02903, USA; alexander_sokolovsky@brown.edu (A.W.S.); judson_brewer@brown.edu (J.A.B.); christopher_kahler@brown.edu (C.W.K.)

**Keywords:** HIV, smoking, mindfulness, technology

## Abstract

Introduction: People with HIV (PWH) who smoke have reported that managing anxiety is a barrier to making a quit attempt and maintaining abstinence post-quit. This study examined the feasibility and acceptability of an app-based mindfulness intervention, *Unwinding Anxiety*, to reduce anxiety prior to a quit attempt in PWH who were not planning to quit in the next 30 days. Methods: Sixteen PWH (mean age 51.5 [SD = 13.2]; mean cigarettes per day 11.4 [SD = 5.4]) were enrolled and followed for eight weeks. A smartphone-based app with 30 modules designed to reduce anxiety was introduced at baseline; participants were encouraged to complete one module daily for four weeks. Symptoms of anxiety and readiness to quit smoking were measured at baseline and weeks 4 and 8. The mean number of modules completed, session attendance, and number of study completers were examined. Generalized estimating equations (GEE) were used to examine changes in self-reported anxiety and readiness to quit at baseline, week 4, and week 8. A brief qualitative interview was conducted at week 4 to explore the acceptability of the app. Results: Feasibility was high, with 93% of participants completing the study. The mean number of study sessions completed was 2.7 (SD = 0.59), and the mean number of modules completed was 16.0 (SD 16.8). Anxiety was high at baseline (M = 14.4, SD = 3.9), but lower at week 4 (b = −5.5; CI: [−9.4, −1.7]; *p* = 0.004) and week 8 (b = −5.1; CI: [−8.8, −1.3]; *p* = 0.008), and stable between weeks 4 and 8 (b = 0.48; CI: [−2.0, 3.0]; *p* = 0.706). Readiness to quit significantly increased from baseline M = 5.5 (SD = 1.6) to week 4 (b = 0.56; CI: [0.20, 0.91]; *p* = 0.002) but was not significantly different from baseline at week 8 (b = 0.34; CI: [−0.30, 1.0]; *p* = 0.30). Ad-hoc moderation analyses found that anxiety had a small significantly positive association with readiness to quit at baseline (main effect: b = 0.10; SE = 0.03; *p* < 0.001) and significantly attenuated the increase in readiness to quit observed at week 4 (anxiety by week 4 interaction: b = −0.08; SE = 0.03; *p* = 0.009). Conclusions: App-based mindfulness training appears to be feasible and acceptable for PWH who smoke and report baseline anxiety. At week 4, anxiety was reduced and readiness to quit was increased, perhaps a key time point for a smoking cessation attempt.

## 1. Introduction

When diagnosed early and engaged in care, people with HIV (PWH) have a near-normal lifespan [1]. However, due to a reported smoking prevalence among PWH of 40–60%, [2] PWH who smoke cigarettes lose more years to smoking than to HIV infection itself [3]. Cancer has emerged as a leading cause of morbidity and mortality in PWH [4,5,6], and a recent study demonstrated that the increased rate of lung cancers in PWH was attributable to cigarette smoking rather than to immunodeficiency or HIV-related factors [6]. Further, it has been estimated that at least 90% of lung cancers and 20% of all other cancers in PWH could be prevented by eliminating smoking [7]. Thus, increasing readiness to quit and improving the success of smoking cessation efforts are key health priorities for this population.

Despite marked decreases in smoking cessation rates in the general population, smoking prevalence in PWH remains high [8], and smoking cessation trials to date have demonstrated disappointing outcomes, with low quit rates and a lack of sustained abstinence [9]. People with HIV who smoke face particular challenges, often reporting high rates of comorbid anxiety and stress, both factors that have been related to poor smoking cessation outcomes. Our qualitative work and that of others has demonstrated that PWH report that smoking helps them manage or alleviate the stresses associated with living with HIV [10,11,12]. Further, they report that stress is a barrier to maintaining abstinence following a quit attempt. While one smoking cessation study demonstrated that one quarter of PWH reported significant anxiety at baseline [13], another found that 80% of PWH reported that anxiety and depressive symptoms were barriers to maintaining abstinence following a quit attempt [14]. Innovative approaches are needed to address anxiety in PWH to reduce symptom frequency and intensity, to reduce a potential barrier to cessation, and to increase readiness to quit smoking. However, few projects to date have addressed the key barrier that anxiety represents to successful smoking cessation in PWH.

Anxiety disorders are common, with a reported lifetime prevalence of approximately 31%, in the general population in the U.S. [15]. Among PWH, a review found that nearly 50% met criteria for moderate to severe anxiety [16]. While only about one third of diagnosed individuals receive treatment for their anxiety disorder, usually in the form of oral medication, many patients, including PWH, are reluctant to add additional medications to their often-extensive treatment regimens [17]. Many individuals may prefer non-pharmacologic therapies [18], and a behavioral treatment, such as mindfulness training (MT), may be especially attractive to them.

Mindfulness has been defined as the awareness that arises when one is paying attention in the present moment, on purpose and nonjudgmentally [19]. When someone is “being mindful”, the attitudinal quality of not judging and allowing their experience to unfold with curiosity helps them avoid being triggered by negative affective states, and to not act out habitual behaviors [20]. In addiction treatment using MT, individuals learn to pay attention, pause, and “be with” their urges, instead of acting on them [21]. Whereas standard smoking cessation treatment typically encourages individuals to distract themselves or substitute another behavior to manage triggers, MT helps people turn toward and manage their thoughts and emotions, thus avoiding a habitual behavior (like smoking).

MT has been shown to be effective for the treatment of mood disorders, such as anxiety [22,23,24,25] and depression [26,27], and thus MT may also be effective in helping PWH quit smoking. MT involves two distinct components: maintaining attention to one’s immediate experience and maintaining an attitude of acceptance toward that experience [28]. Through these complementary components of present-centered awareness and acceptance, MT targets the negative reinforcement processes of compulsive smoking. People who smoke may be able to learn to pay attention to and accept affective states (such as anxiety and craving) as they arise, rather than reacting by smoking a cigarette [29].

There have been few well-designed adequately powered randomized studies examining the use of MT for smoking cessation [30]. Preliminary studies have shown utility in reducing cigarette cravings and withdrawal symptoms [31], as well as improvements in smoking cessation rates [32,33]. Bowen and colleagues provided college students (N = 123) with brief MT and found that they smoked significantly fewer cigarettes one week post-intervention than those who did not receive the training [34]. Davis and colleagues found 10 of 18 subjects maintained abstinence 6 weeks post-quit after mindfulness-based stress reduction [32]. Brewer and colleagues randomized 88 subjects to MT or the American Lung Association Freedom From Smoking program [33]. Subjects randomized to MT had significantly greater reductions in smoking at the 4-month follow-up (*F* = 11.11, *p* = 0.001). The MT group also had significantly greater 7-day point prevalence abstinence post-treatment (36% vs. 15%, *χ*^2^ = 3.5, *p* = 0.063) and at a 17-week follow-up (31% vs. 6%, *χ*^2^ = 6.32, *p* = 0.012). MT practice significantly predicted smoking reduction (daily meditation: *β* = −1.21, *p* = 0.007; informal on-the-go practices: *β* = −1.52, *p* < 0.0001) and moderated the relationship between craving and smoking (*β* = 0.515, *p* = 0.026) [35]. In this pilot trial, the more that individuals practiced mindfulness, the less they smoked, and the amount of practice predicted the decoupling of craving and smoking. While these small studies have demonstrated the preliminary efficacy of MT for smoking cessation, a meta-analysis found that MT did not significantly differ from comparator conditions, such as cognitive behavioral therapy (CBT), in their effect on cessation [36].

PWH who smoke have reported that anxiety is a significant barrier to making a quit attempt. Therefore, addressing anxiety prior to making a quit attempt may improve overall smoking cessation outcomes. The primary aim of this study was to examine the feasibility, acceptability, and preliminary efficacy of a MT app, *Unwinding Anxiety*, to reduce anxiety in PWH who smoke. Secondly, we aimed to examine whether reduction in self-reported anxiety was associated with increased readiness to quit smoking. We hypothesized that participants who had greater reductions in anxiety would have increased readiness to quit smoking.

## 2. Methods

### 2.1. Participants

Sixteen PWH, who endorsed an overall desire to quit smoking but not in the next 30 days, were recruited and enrolled online via Zoom. To be eligible, participants had to be diagnosed with HIV, age 18 years or older, fluent in English, smoke cigarettes daily, own a smartphone with internet access, and score 10 or more on the generalized anxiety disorder scale (GAD-7) [37], to indicate at least a moderate level of anxiety. It was hypothesized that people with minimal anxiety would be less engaged in the intervention. Exclusion criteria included intention to quit smoking in next 30 days (score of ≥7 on the contemplation ladder [38]), current use of smoking cessation pharmacotherapy, an unstable medical or psychiatric condition (defined as hospitalization in prior 30 days), psychotic symptoms, substance use disorder other than nicotine dependence, past-month suicidal ideation or past-year suicide attempt, and pregnant/nursing.

Participants were recruited from December 2020 through August 2021. Study flyers were posted on clinic bulletin boards, in the Providence community, at AIDS-service organizations, on Craigslist, and on social media platforms such as Facebook. Study flyers said, “Are you a smoker with HIV? Do you sometimes struggle with anxiety and stress? We are examining a new way to help smokers manage stress”. Potential participants tele-phoned in response to the ad, and were screened for eligibility by the research assistant. Those who were eligible based on the initial phone screening were invited to schedule a baseline interview. All the study sessions were conducted online via Zoom.

### 2.2. Study Design

Participants attended an online baseline (BL) study visit in which behavioral and subjective assessments were collected. A NicConfirm nicotine-detection saliva test was mailed to the participant’s home prior to the scheduled BL visit and a positive test during the Zoom session was required as evidence of smoking status. The study research assistant (RA) helped participants download the *Unwinding Anxiety* app, oriented them to its functionality, and instructed them to start using the app that day. Participants were encouraged to complete one module daily but were given 8 weeks to complete 30 modules. Participants received a follow-up phone call 2 days after the BL session to correct or assist with any difficulties in using the app. Following the BL study session, participants were scheduled for online study appointments via Zoom at weeks 4 and 8. Participants received up to $100 in compensation for their time: $30 for the BL; $30 for the week 4 assessment, and $40 for the week 8 assessment. Compensation was not provided for the nurse treatment session and was not contingent upon smoking status.

#### Ethical Considerations

All study participants completed the informed consent process conducted online via Qualtrics prior to any data collection. The study procedures were approved by the Brown University Institutional Review Board.

### 2.3. Intervention

MT was delivered through an app-based platform, *Unwinding Anxiety*, which has been described previously [39]. Briefly, it includes 30 core modules of brief didactic and experience-based MT (videos and animations, roughly 10 min/day), guided meditations (5–15 min), app-initiated check-ins, and brief on-demand mindfulness exercises to help with anxiety. The intervention content is based on a framework from previously developed in-person and app-based MT programs that share a core underlying mechanism [33,40].

### 2.4. Baseline Visit

During the BL study appointment, study procedures, potential risks, and compensation were discussed. Those who were interested completed an informed consent form via Qualtrics. Participants then completed an interview to confirm eligibility and complete all the baseline measures via Qualtrics. Participants were assessed on a variety of interview and self-reporting measures.

### 2.5. Follow-Up Assessments at Weeks 4 and 8

Participants were scheduled for follow-up online assessments at 4 and 8 weeks following the BL session. At these sessions, participants completed questionnaires and surveys. Participants were asked at week 4 if they would like to set a quit date.

#### Week 4 Brief Qualitative Interview

A brief qualitative interview was conducted by the research assistant at the conclusion of the week 4 follow-up visit. The interview guide was developed a priori and included six questions to explore participants’ perceptions of the usability and helpfulness the *Unwinding Anxiety* app that was utilized in the study. The interviews were audio recorded and transcribed by the principal investigator (PI). The transcribed interviews were coded by hand by the PI and themes emerged. The extracted themes were reviewed by the study team (PI and co-investigators). Illustrative quotes to support the themes were extracted by the PI from the transcriptions. Consensus was reached within the study team on the appropriateness of the themes and quotes.

### 2.6. Setting a Quit Date

Smoking cessation was not addressed at the BL session to allow participants to focus on MT and anxiety management. At the week 4 session, readiness to quit was assessed using a standardized scale (contemplation ladder score ≥ 7). If a participant expressed a willingness to set a quit date in the next 30 days, an online treatment session was scheduled with the study nurse. At that 30 min session, standard smoking cessation counseling was provided and a 2-week supply of nicotine replacement therapy (dose based on current level of smoking) and resources for quitting (smokefree.gov; Quitline) were mailed to their residence. A nurse telephone call was scheduled for their quit date and one week after their quit date. Participants were instructed to continue to use their app and practice their mindfulness skills to manage anxiety symptoms during the quit attempt. They were instructed to obtain further NRT through their health care provider and health insurance.

## 3. Measures

### 3.1. Feasibility and Acceptability

Feasibility and acceptability measures included the number of modules completed. The app has the capability to monitor and track usage and uptake of the app and its modules. We also examined session attendance (recorded as a continuous variable ranging from 1 to 3 online study sessions) and retention (coded as a dichotomous variable with participants who complete the 8-week online session characterized as completers and those not attending the final online session as non-completers). The system usability scale (SUS), a 10-item Likert-type scale, used to assess the usability of software, websites, and applications [41], was administered at week 8. It has been used widely and has good validity and reliability. An average SUS score is 68, with scores above 68 considered above average and those under 68 considered below average.

#### Brief Qualitative Interviews

We asked the following questions as part of the brief qualitative interview at week 4:

“*Please tell us what you liked (or disliked) about using the Unwinding Anxiety app. In general, did you find it helpful in managing your anxiety? If yes, in what ways was it helpful? If no, why do you think it was not helpful? On average, how often did you practice mindfulness once you started using the app? What improvements or changes might you make to the app to make it more helpful to manage your anxiety?*”

### 3.2. Anxiety

The generalized anxiety disorder scale (GAD-7), a self-reported questionnaire for measuring the severity of GAD [37], was administered at BL, and at weeks 4 and 8. The GAD-7 demonstrated good to excellent reliability (Cronbach’s alpha: BL, 0.65; week 4, 0.94; week 8, 0.93).

### 3.3. Smoking Measures

The Fagerström test for cigarette dependence (FTCD) was administered to evaluate severity of cigarette dependence at BL. The contemplation ladder [38] assessed readiness to quit smoking at BL, and at weeks 4 and 8. Smoking cessation self-efficacy was assessed using a 1-item Likert scale (range 1–10) that asked about confidence to quit permanently and was measured at BL, and at weeks 4 and 8. Timeline followback (TLFB) was used to assess cigarettes smoked per day (CPD), use of nicotine replacement therapy, 7-day point prevalence abstinence, and sustained abstinence (defined as no cigarette smoking since their quit date) at week 8. To measure quit attempts, participants were asked at weeks 4 and 8 if they had had a period of no smoking for longer than 24 h since their last study session.

## 4. Data Analysis Plan

Preliminary analyses included computing descriptive statistics (Aim 1) and evaluating the distribution properties of baseline variables and correlations among outcome measures. We used generalized estimating equations (GEE) specifying an autoregressive correlation structure (AR1) and using a discontinuous (i.e., categorical) measure of time to examine within-person change in anxiety and readiness to quit (Aim 2). We fit one further GEE model to examine whether anxiety moderated the within-person change in readiness to quit over time (Aim 3). We computed 95% confidence intervals (CIs) for all estimates. Descriptive analyses were conducted in SPSS version 24. All the GEE models were fit in R 4.1.2 [42] using the geepack package [43].

## 5. Results

### 5.1. Sample Characteristics

Sixteen PWH who smoked daily and who endorsed a desire to quit smoking, but who were not planning to do so in the next 30 days, were enrolled. One participant did not complete the baseline session with the app training and was withdrawn by the PI. Analyses were based on the 15 participants that completed the entire baseline session. At BL, the mean age was 51.5 (SD = 13.2), 7 (46.7%) of the participants smoked 10 or less CPD, while 8 (53.3%) participants smoked 11–20 CPD. The mean CPD was 11.4 (SD = 5.4). The complete demographic and clinical characteristics of the sample are shown in Table 1.

### 5.2. Feasibility and Acceptability

Fourteen (93.3%) participants completed the study, demonstrating very good feasibility. The mean number of study sessions completed was 2.7 (SD = 0.59) out of a possible 3 sessions. Twelve participants completed the week 4 session, while 14 completed the final (week 8) session. Twelve participants (80%) completed all three study sessions. The mean number of app modules completed was 16.0 (SD 16.8) out of a possible 30. Two participants completed all 30 modules, while one participant completed each module twice (total of 60 modules). The mean SUS score was 81.4 (SD = 24.2), with a median score of 87.5, indicating excellent app usability.

### 5.3. Change in Anxiety, Readiness to Quit, and Self-Efficacy for Smoking Cessation

**Figure 1:**
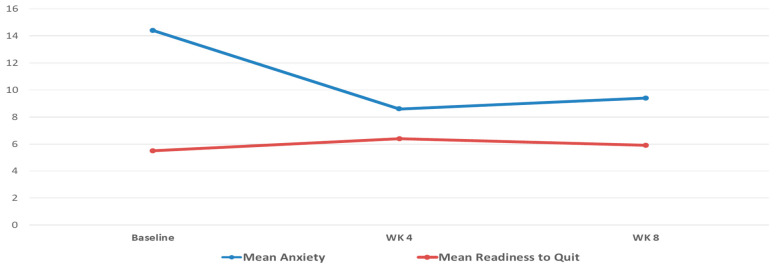
Mean Anxiety and Readiness to Quit at Baseline, Week 4, and Week 8.

Anxiety was high at baseline (M = 14.4, SD = 3.9), but significantly lower at week 4 (b = −5.5; CI: [−9.4, −1.7]; *p* = 0.004) and week 8 (b = −5.1; CI: [−8.8, −1.3]; *p* = 0.008) relative to baseline, and stable between weeks 4 and 8 (b = 0.48; CI: [−2.0, 3.0]; *p* = 0.706). Readiness to quit increased significantly from baseline (M = 5.5, SD = 1.6) to week 4 (b = 0.56; CI: [0.20, 0.91]; *p* = 0.002) but was not significantly different from baseline at week 8 (b = 0.34; CI: [−0.30, 1.0]; *p* = 0.30). See Figure 1.

Ad hoc moderation analyses found that anxiety had a small positive association with readiness to quit at baseline (b = 0.10; CI: [0.046, 0.15]; *p* < 0.001), and greater anxiety was associated with significantly less increase in readiness to quit at week 4 (interaction effect: b = −0.08; CI: [−0.14, −0.020]; *p* = 0.009). Self-efficacy for smoking cessation was stable and did not significantly change over time, with a mean of 6.42 (SD = 0.99) at BL, 5.86 (SD = 2.0) at week 4, and 5.53 (SD = 1.5) at week 8.

### 5.4. Relationship between Engagement, Anxiety, and Readiness to Quit

At week 8, engagement was not significantly correlated with either anxiety (r = −0.27, *p* = 0.37) or readiness to quit (r = 0.53, *p* = 0.06).

### 5.5. Quit Outcomes

At week 4, one participant indicated that they were ready to quit in the next 30 days. A meeting with the nurse was completed and the participant set a quit date.

At the week 8 study visit, three participants (20%) reported making a quit attempt, and 3 (20%) self-reported that they had picked up a prescription for nicotine replacement therapy. Two participants (13.3%) self-reported 7-day point prevalence abstinence. One participant self-reported sustained abstinence for 23 days, while a second had had a sustained abstinence of 20 days at the week 8 study session.

### 5.6. Qualitative Findings

Two major themes emerged from the qualitative interviews:

I. *Acceptability of Unwinding Anxiety.*

Illustrative quotes that were extracted from the qualitative interviews supported the acceptability of the *Unwinding Anxiety* app. Participants indicated that the *Unwinding Anxiety* app was useful in that it helped them manage and reduce their symptoms of anxiety. Most participants indicated that they used the app and practiced the MT exercises daily. The following quotes illustrate these points:

*(I) used it more often than daily. [I] used it for anxiety before bed. It helped me clear my mind, using it then. I would focus on, like, how I was laying/the position I was laying in. I would focus on what hurt on me/what was causing me the anxiety, and then it brought me to the point of actually sleeping.*

(901)

*I like the app. I do. The videos that are on there, some of them are really interesting. They teach me how to cope with stuff. Like breathing habits. I like how it lets you check in to see where you are, how you’re feeling. It just taught me different techniques on how to deal with everything. Pretty much, the breathing. And that really helps a lot… The checking in is my favorite part, I think. I’m focusing on it. It helps me zone in and focus on what I’m doing [breathing]. When I am anxious, if I tell myself to breathe, like now, I know I can zone in now right on my breathing. And everything else just kinda flurries away.*

(902)

*My anxiety was kicking up, and then I did the breathing, I went right to the app, and I started doing the stress test to see where I was at, and it showed I was a little high, so I started focusing on the breathing and I did the walking. So what is my body doing? it taught me the different feelings that I’m having in my body at that time. So the next day, I started breathing and paying attention and finding out where this anxiety was coming from. That was awesome to me. Your body tells you a lot. I’m learning… a lot of things to pay attention to… and it’s helped me a lot.*

(910)

II. *Need for customization or tailoring of app.*

When asked what they disliked about the app or improvements they might recommend, some participants indicated that they wished they could have completed more than one module daily, saying:

“*I disliked how once you finish a module you can’t go forward to the next one. I’m the type of person that if I’m interested in something and I’m zoned in on it, I just want to keep going. And the fact that it stops me, that’s aggravating.*”

(902)

Another participant expressed a similar point:

*The main thing is I’m doing the module and it ends and it says, “good job”. (I don’t like that). I want to just keep moving on and it won’t let me.*

(910)

## 6. Discussion

To our knowledge, this is the first study to examine the feasibility, acceptability, and preliminary efficacy of an app-based delivery of MT to reduce anxiety in PWH who smoke. Overall, this study showed promising results. Study participation and retention rates were high, demonstrating excellent feasibility for conducting a larger trial of app-based MT in this population. Acceptability of the *Unwinding Anxiety* MT app was high both in terms of number of modules completed, and the scores on the SUS. Participant statements from the qualitative interviews further supported both the usability of the *Unwinding Anxiety* app and the helpfulness of the mindfulness exercises to manage anxiety. In addition, at week 4, anxiety levels were significantly reduced and readiness to quit was increased, perhaps suggesting that 4 weeks of app-based MT for anxiety reduction may be a reasonable duration and that week 4 may be a key timepoint for a cessation attempt.

Anxiety disorders are common among PWH and a recent study of PWH who used substances reported that approximately one-third (31.4%) of the sample reported experiencing moderate/severe symptoms of generalized anxiety disorder (GAD) [44]. Further, after controlling for covariates, participants with moderate/severe symptoms of GAD had nearly twice the odds of being current smokers (AOR = 1.70, 95% CI = 1.18–2.45 *p* = 0.004), so addressing both anxiety and cigarette smoking seems to be a reasonable approach [44]. Mindfulness-based interventions have been shown to be effective in reducing anxiety among other populations living with chronic illness, such as cancer [45]. It has also been shown to be effective for outpatients who did not respond sufficiently to medication treatments and other psychological treatments [46]. MT may hold promise for treating PWH with anxiety, who are often resistant to adding additional medications to their already complex regimens.

Our study demonstrated a significant association between lower anxiety and increased readiness to quit smoking among PWH after 4 weeks of mindfulness training. This would seem to suggest that a sequential therapy for PWH focused on anxiety reduction initially, followed by smoking cessation treatment, may improve smoking cessation outcomes. A study in polysubstance users in the general population demonstrated that mindfulness-based stress reduction was associated with increases in self-efficacy over time, which predicted a significantly higher probability of no drug use and no heavy drinking at the 12-month follow-up [47,48]. While we did not find a significant change in self-efficacy for smoking cessation, we did see significantly increased readiness to quit smoking. Interestingly, however, only one participant indicated a willingness at week 4 to meet with the study nurse and set a quit date. This may suggest that using an opt-out approach may be required to further motivate PWH who smoke to make a quit attempt. This approach has been shown to be effective in this population [49].

This study has both strengths and limitations. To our knowledge, it is the first study to examine the feasibility and acceptability of app-based mindfulness training to reduce anxiety symptoms in PWH who smoke. A major limitation of this study is its small sample size and lack of a control group. Our findings may not be representative of all people living with HIV. Further study with larger samples and randomization is clearly needed in this priority population of smokers. Our participants were all above 30 years of age, so it is unclear whether these findings would be applicable to a younger age group. We only recruited people with HIV who spoke English. Future studies should recruit a more diverse sample with non-English-speaking participants. Finally, we found that app engagement was not significantly correlated with either anxiety or readiness to quit at week 8, and this requires further exploration.

## 7. Conclusions

In summary, app-based mindfulness training appears to be feasible and acceptable for PWH who smoke and report anxiety as a barrier to quitting. At week 4, anxiety was reduced and readiness to quit was increased, perhaps a key time point for a cessation attempt. MT may be a promising means of reducing anxiety, and further study could examine whether the increased readiness to quit results in greater uptake of smoking cessation treatment. A full-scale randomized trial to examine whether mindfulness for anxiety reduction prior to a quit attempt will improve quit rates and sustained abstinence in PWH is recommended.

## Figures and Tables

**Table 1:** Sample characteristics at baseline (n = 15).

Variable	Mean (SD)	Range
Age	51.5 (13.2)	30–67
Cigarettes per day	11.4 (5.4)	3–20
FTCD score	6.0 (1.6)	2–8
Number of lifetime quit attempts	5.3 (6.7)	0–25
Number of years smoked	35.0 (13.7)	15–54
Mean Anxiety score (GAD-7)	14.4 (3.9)	9–21
**Variable**		**n (%)**
Race		
White		8 (53.3)
Black or African American		5 (33.3)
Other		2 (13.3)
Ethnicity		
Hispanic/Latinx		6 (40.0)
Employment Status		
Employed, part/full-time		3 (20.0)
Unemployed		1 (6.7)
Disabled		7 (46.7)
Retired		4 (26.7)
Education		
Some high school		4 (27)
High school/some college		10 (67)
College Graduate or Higher		1 (6)
Sex Assigned at Birth		
Female		6 (40)
Male		9 (60)
Gender, self-identified		
Female		7 (47.0)
Male		8 (53.3)
Transgender female		1 (6.7)

## Data Availability

De-identified data may be available by written request to the Corresponding Author.
